# Skin regeneration-related mechanisms of Calcium Hydroxylapatite (CaHA): a systematic review

**DOI:** 10.3389/fmed.2023.1195934

**Published:** 2023-06-02

**Authors:** Mojgan Amiri, Renald Meçani, Christa D. Niehot, Terri Phillips, Janina Kolb, Hua Daughtry, Taulant Muka

**Affiliations:** ^1^Department of Epidemiology, Erasmus MC University Medical Center, Rotterdam, Netherlands; ^2^Epistudia, Bern, Switzerland; ^3^Division of Endocrinology and Diabetology, Department of Internal Medicine, Medical University of Graz, Graz, Austria; ^4^Literature Searches Support, Dordrecht, Netherlands; ^5^Merz North America, Inc., Raleigh, NC, United States; ^6^Merz Aesthetics GmbH, Frankfurt, Germany; ^7^Institute of Social and Preventive Medicine (ISPM), University of Bern, Bern, Switzerland

**Keywords:** Calcium Hydroxylapatite (CaHA), skin regeneration, mechanism, elastic fiber, angiogeneisis

## Abstract

**Introduction:**

Calcium Hydroxylapatite (CaHA) is a common dermal filler used in aesthetic medicine for volumizing and contouring. Understanding mechanisms of actions of CaHA can help improve our understanding of its clinical applications.

**Methods:**

We performed a systematic review to summarize the skin-regeneration related mechanisms of CaHA. Five bibliographic databases were searched for English-language publications that evaluated CaHA in skin regeneration outcomes including neocollagenesis, cell proliferation and growth factors, angiogenesis, vascular dynamic and inflammatory markers, among others. Methodological rigor of included studies was assessed.

**Results:**

Of 2,935 identified citations, 12 studies were included for final analysis. Collagen production was reported by nine studies, cell proliferation by four, elastic fibers and/or elastin by four, and three studies on angiogenesis, while limited studies were available on the other outcomes. Six were clinical/observational studies. Only seven studies had a control group. Overall, studies showed CaHA resulted in increased cell proliferation, increased collagen production and angiogenesis, as well as in higher elastic fiber and elastin formation. Limited and inconclusive evidence was available on the other mechanisms. The majority of the studies had methodological limitations.

**Discussion:**

Current evidence is limited but indicates several mechanisms through which CaHA could lead to skin regeneration, volume enhancement, and contouring.

**Systematic review registration:**

https://doi.org/10.17605/OSF.IO/WY49V.

## Introduction

Demands for minimally invasive rejuvenative procedures to counteract age-related loss in youthful appearance and signs of aging such as skin wrinkles, deep furrows, and bone resorption, especially in the face, are increasing. Calcium Hydroxylapatite (CaHA) is a common minimally invasive dermal filler used to ameliorate certain skin aging signs providing “real-time” results with good outcomes and high patient satisfaction scores. In addition, evidence shows that CaHA in general has a good safety profile (1). While several CaHA fillers exist, Radiesse® (Merz North America, Inc., Raleigh, NC, USA) is currently the only CaHA filler approved by FDA for facial augmentation.

Skin aging is a common phenomenon in which the skin can no longer maintain normal thickness, strength, function, and hair density. By affecting different cell mechanisms, such as collagen synthesis, elastin production and angiogenesis, it is possible to stimulate regeneration in senescent tissue, an emerging concept referred to as regenerative aesthetics (2). Several such mechanisms have been suggested to support CaHA as a regenerative aesthetic treatment. Studies have shown that CaHA can lead to proliferation of different cells, increased collagen and elastin production, as well as increased number of new vascular beds (1). Nevertheless, studies have used different markers for the evaluated mechanisms, and yield variable results (3–7). Furthermore, studies have used different time-points, and therefore uncertainty remains on the time-interval during which CaHA fillers affect the mechanisms described. Understanding mechanisms through which CaHA exerts its regenerative effects on skin can help enhance our understanding of CaHA and its uses in aesthetic medicine.

We performed a systematic review to summarize the evidence on skin-regeneration related mechanisms affected by CaHA.

## Methods

### Review design

The review was conducted following an established guide on conducting systematic reviews and meta-analyses for medical research and reported based on PRISMA guidelines (Appendix 1) (8). Its protocol has been registered with the Open Science Framework (https://doi.org/10.17605/OSF.IO/WY49V). The current review is part of a large project, with detailed information specified in the mentioned protocol.

### Data sources and search strategy

Four electronic databases, including Embase via Embase.com, Ovid via Medline, Web of Science via Clarivate, and Cochrane Central Register of Controlled Trials via Wiley were searched from inception until October 4, 2022 (date last searched). Additionally, the first 200 results from the Google Scholar search engine were downloaded using Publish or Perish software [Harzing, A.W. Publish or Perish. 2007. Available online: https://harzing.com/resources/publish-or-perish (accessed on October 4 2022)]. A single search string query was used to search the databases for reproducibility and adaptability. During the search, the queries were refined to exclude conference abstracts, systematic reviews and meta-analyses. The following element/concept was used: Radiesse and CaHa. The results were deduplicated using the Bramer/Erasmus MC method in EndNote (9). No authors or subject experts were contacted. The complete search strategy can be found in Appendix 2. The search strategy was developed by an expert research librarian. Furthermore, we manually screened the references of the final included studies for additional relevant studies.

### Inclusion and exclusion criteria

For the current research question, we included studies of any study design that (i) were conducted in animals, humans, or *in vitro*; (ii) assessed and applied CaHA for dermatological/aesthetic purposes; and (iii) evaluated outcomes that relate to mechanisms of dermal regeneration including neocollagenesis, cell proliferation and growth factors, angiogenesis, vascular dynamic (e.g., vasodilatation, vasoconstriction, venoconstriction, vascularization) and inflammatory markers, among others. Case reports of <10 participants, reviews, letters to editors, and abstracts were included. We excluded studies that use CaHA for purposes other than aesthetics such as use of CaHA in bone tissue for bone regeneration.

### Study selection, data extraction, and quality assessment

Two reviewers independently assessed titles and abstracts, and afterwards evaluated the full texts for eligibility. The data extraction was performed by one of the authors and the extracted data was further checked by other authors. Any disagreement between reviewers was settled by reaching a consensus or by consulting a third reviewer. Authors extracted data on name of first author, year of publication, study design, tissue or animal used, number of subjects included in the studies, follow-up, type, and characteristics of the skin filler (e.g., brand, dosage), outcome and assessment methods, and the results/findings (including measure of associations reported). Risk of bias was assessed by different methods, depending on the study design and whether it was conducted in humans; a summary of the checklists used can be found in Appendix 3.

### Statistical analysis

Due to high heterogeneity in the designs, subjects included, outcomes, and metrics, we provided a descriptive analysis. For each study, we reported the effect magnitude, direction, and significance. We constructed separate tables for the methods used to assess the outcomes, main findings, and methodological appraisal of included studies.

## Results

### Study characteristics

**Figure 1:**
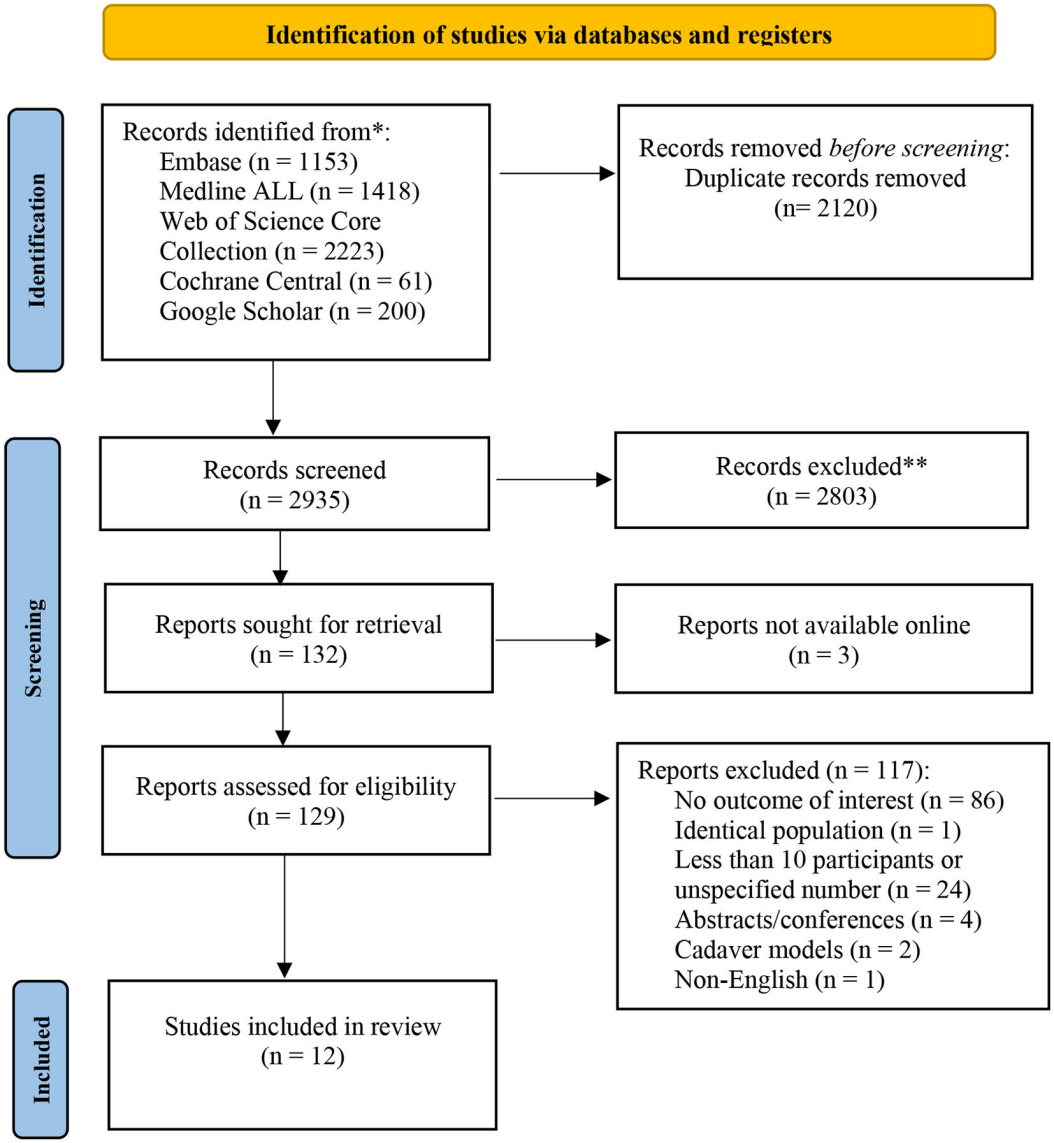
Flowchart of identification, screening, eligibility, inclusion, and exclusion of retrieved studies.

Of 2,935 unique citations generated from the search strategy, 128 full-text articles were retrieved for full text assessment. Of those, only 12 studies met our inclusion criteria, and were therefore included for final analysis (Figure 1). Characteristics of the included studies are provided in Table 1 and Appendix 4. Six studies (5, 6, 13, 15, 16) were conducted in human subjects; of those, three were single-arm intervention studies, one was a paired-design clinical study, one, a retrospective study, and one a prospective cohort study, with the number of subjects varying from 15 to 92. Only two studies (6, 7) included males, while the rest were focused only on women. The most common injection site was the face, with only two studies (13, 16) performed on the neck and hand areas. One study involved HIV-positive patients with facial lipoatrophy (7). Five studies used lidocaine as part of the dilution technique (5–7, 15, 16). In none of the studies, did any participant receive any additional simultaneous treatments with CaHa. The maximum average follow-up was 12 months.

**Table 1:** General Characteristics of included studies.

**Author, year**	**Country**	**Cell/Animal type**	**Age**	***In Vitro*, *Vivo*, Randomized**	**No. of groups**	**No. of participants (overall and per group)**	**Follow-up time**	**Intervention**	**Control**	**Outcome**
Wollina et al. (10)	Germany	Human HaCaT keratinocytes and human dermal fi-broblasts	NA	*In vitro*	NP	NP	1 to 48 h	Radiesse 3.0 cc	HaCaT keratinocytes or human dermal fibroblasts grown in DMEM	• Cell proliferations •Cytotoxic effects
Yanatma et al. (11)	Turkey	Winstar albino rats (Female)	1 y (Average age)	Experimental	3	30/ 10	2 and 4 m	CaHA, no brand specified	• Group 1: polycaprolactone; •Group 2: no intervention	• Total collagen Density •Dermis thickness and cell count •Collagen I and III •Collagen I and III immunoreactivity levels of fibroblasts
González et al. (5)	USA	Individuals (Women)	35-60 y	One-arm intervention	1	15/15	6 m	CaHA, no brand specification	No control	• Elastic fibers •Elastin •Proteoglycan
Hwang et al. (12)	South Korea	A human adult dermal fibroblast (hADF) cell line was and skin-wrinkled mouse model; SKH1-hairless mice (Male)	Mouse: 5 w	*In vitro* and *in vivo*	5	Cell experiment, n=4	Animal experiment: 8 w	HAp (0.1–5 wt%, Radiesse)	• Cell experiment: Cell only without hydrogel •Animal experiment: VD3 (positive control).	• Cell proliferation •Anti-wrinkle efficacy, collagen production
Fan et al. (4)	South Korea	BALB/c nude mice	5 w	Experimental	2	24/ 20 and 4	12 w	HAp (Radiesse, Raleigh, North Carolina)	HAc (Restylane, Upp- sala, Sweden), HAc-nano-HAp, or HAc-micro-Hap and Uninjected mice	• Gene expression and protein level •Collagen and elastic fiber synthesis
Rovatti et al. (6)	Italy	Individuals (Men/Women)	57.1 ± 9.3 y (38–72)	Retrospective study	1	40	4 m post treatment	Hyperdiluted CaHA1:2, no brand provided	No	• Collagen morphology •Vessel density
Yutskovskaya et al. (13)	Russia	Individuals (Women)	39.8 ± 3.97 y	One arm intervention	1	20	4 m	Diluted CaHA, MERZ: 1:2 dilution for normal skin; 1:4 dilution for thin skin and 1:6 for atrophic skin	No	• Collagen I and III expression •Elastin and angiogenesis
Coleman et al. (14)	USA	Animal, not specified type	NS	One-arm experimental	1	6	78 w	CaHA, Radiesse	No	• Neocollagenesis
Yutskovskaya et al. (15)	Russa	Individuals (Women)	34–45 y	Randomized, split-face, comparative clinical study; paired design	2	24/24	9 m ± 2 w	CaHA gel matrix	HA gel	• Collagen expression •Elastin expression •Angiogenesis •Lymphohistiocytic infiltration •Mucoid edema •Ki-67 Staining
Courderot-Masuyer et al. (3)	France	Wrinkle fibroblasts and normal aged fibroblasts from facelifts (Women)	49–55 y	*In vitro*	1	3/3	NA	CaHA Radiesse	TGFb1	• Contractile forces
Figueredo et al. (16)	Brazil	Skin biopsies (3-mm punch) from the dorsum of the hands (Women)	53.2 ± 5 y	One-arm experimental	1	13	24 w	CaHA, Radiesse	No control	• Total collagen density •Collagen I and III
van Rozelaar et al. (7)	Netherlands	One or more facial sites in a fan-like manner in men and women with HIV	Range 31–72 y	Prospective cohort	2	82/ 41 each group	12 months	CaHA	Poly-L-lactic acid (PLLA)	• Collagen formation •Inflammation

NP, not provided; NA, not ascertain???; h, hours; w, weeks; m, months; y, years.

Of the non-human studies, three studies were *in vivo*/experimental studies (4, 11, 14), two (3, 10) were *in vitro* and one study (11) presented both *in vivo* and *in vitro* data. Dermal fibroblasts were used in three *in vitro* studies. Among the experimental animal studies, 2 studies used mice, one rat and the other did not specify the animal, type. Eight studies (3, 4, 10, 12–16) used Radiesse™ as the CaHA filler, while the other studies (5–7, 11) did not specify the brand of the CaHA.

Concerning outcomes, four studies reported on cell proliferation, nine studies on collagen production, four studies on elastic fibers and/or elastin, three studies on angiogenesis, two on inflammation, while proteoglycan, gene and protein expression were reported by only one study. Methods used to assess the outcomes in each study are summarized in Appendix 5. Main findings from individual studies are summarized in Table 2.

**Table 2:** Main findings from the included studies.

**Author, year**	**Outcome**	**Intervention baseline**	**Intervention after**	**Difference before/after**	**Control baseline**	**Control after**	**Control before/after**	**Intervention vs. control**
Wollina et al. (10)	Cell proliferation, ATP	NP	NP	NP	NP	NP	NP	Cell proliferation and cell viability were not significantly changed by CaHA. ATP concentrations were slightly higher in human dermal fibroblast cultures. ATP concentration increased with time of exposure from 1 h to 48 h in the negative control, with the probes indicating no negative effect of CaHA
	Cytotoxicity	NP	NP	NP	NP	NP	NP	LDH concentration did not increase with time of exposure. There was no linear dose-dependency either. Cytotoxicity study did not show a dose-dependency or an exposure-time dependency. There was only a slight dose-dependent increase of LDH
Yanatma et al. (11)^*^	Collagen density	NP	2 months: 76.63 (69.59-80.26) 4 months: 76.85 (71.78–87.42)	No difference, *p =* 0.779	NP	• PCL group, 2 months: 72.25 (70.73–93.97) •PCL group, 4 months: 76.34 (66.68–90.08) •No intervention Group, 2 months: 67.73 (59.61–77.14) •No intervention Group, 4 months: 68.79 (57.01-76.04)	• PCL Group: no difference, *p =* 0.594 •No intervention group: no difference, *p =* 0.515	Both at 2 months (*p =* 0.002) and at 4 months (*p =* 0.024) there was an increase in collagen density in CaHA and PCL group compared to no intervention group, but no difference was found between CaHA and PCL groups
	Dermis thickness (μm)	NP	2 months: 600.45 (398.47–55 658) 4 months: 579.37 (458.68-623.19)	No difference, *p =* 0.674	NP	• PCL group, 2 months: 490.23 (428.35–623.01) •PCL group, 4 months: 590.51 (405.54-736.38) •No intervention Group, 2 months: 526.08 (454.49–590.51) •No intervention Group, 4 months: 411.77 (368.92–505.41)	• PCL Group: no difference, *p =* 0.066•No intervention group: decrease, *p =* 0.017	• 2 months: No significant difference was found between any groups (*p =* 0.325). •4 months: there was a significant difference in both the PCL and CaHA groups compared to the control group (p < 0.001). However, there was no difference between PCL and CaHA groups.
	Number of fibroblast nuclei	NP	2 months: 33 (26–42) 4 months: 32 (27–43)	No difference, *p =* 0.866	NP	• PCL group, 2 months: 33 (27–40) •PCL group, 4 months: 39 (32–64) •No intervention Group, 2 months: 29 (21-41) •No intervention Group, 4 months: 28 (23-34)	• PCL Group: increase, *p =* 0.007 •No intervention group: no difference, *p =* 0.553	• 2 months: no significant difference between groups, *p =* 0.494 •4 months: there was a difference between the three groups, with PCL group having higher number (*p* < 0.001)
	Type 1 collagen H score	NP	2 months: 210 (160–270) 4 months: 210 (160-270)	• No difference, *p =* 1	NP	PCL group, 2 months: 210 (160–270) •PCL group, 4 months: 240 (160–270) •No intervention Group, 2 months: 120 (50–160) •No intervention Group, 4 months: 140 (60–160)	• PCL Group: no difference, *p =* 0.478•No intervention group: no difference, *p =* 0.671	• 2 months: Type 3 collagen H scores increased significantly in both the PCL and CAHA groups in the compared to the control group (*p* < 0.001). However, there was no difference between the two filler groups. •4 months: Type 3 collagen H scores increased significantly in both the PCL and CAHA groups in the compared to the control group (*p* < 0.001). However, there was no difference between the two filler groups.
	Type 3 collagen H score	NP	2 months: 180 (160–270) 4 months: 180 (160–240)	No difference, *p =* 0.248	NP	• PCL group, 2 months: 240 (160–270) •PCL group, 4 months: 240 (140–270) •No intervention Group, 2 months: 140 (70–160) •No intervention Group, 4 months: 120 (70–160)	• PCL Group: no difference, *p =* 0.786•No intervention group: no difference, *p =* 0.944	• 2 months: Type 3 collagen H scores increased significantly in both the PCL and CAHA groups in the compared to the control group (*p =* 0.001). However, there was no difference between the two filler groups. •4 months: Type 3 collagen H scores increased significantly in both the PCL and CAHA groups in the compared to the control group (*p* < 0.001). However, there was no difference between the two filler groups.
	Type 1/Type 3 collagen H score	NP	2 months: 1.0 (0.89–1.33) 4 months: 1.0 (0.67–1.69)	NP	NP	• PCL group, 2 months: 1.0 (0.67–1.69) •PCL group, 4 months: 1.0 (0.88–1.50) •No intervention Group, 2 months: 0.86 (0.36-1.75) •No intervention Group, 4 months: 1.0 (0.50-1.17)	NP	• 2 months: no difference between groups, *p =* 0.355 •4 months: no difference between groups, *p =* 0.691
González et al. (5)	Elastic Fibers	Mean 24%, SE 5.65	Mean 19.71%, SE 3.53	Mean change −17.88, SE −37.18, *p =* 0.528. Some subjects showed an increase in elastin between 12% and 44% from baseline. The changes overall in the group as a whole were not statistically significant.	NA	NA	NA	NA
	Elastin	Mean 6.32%, SE 1.14	Mean 4.35, SE 0.84	Mean difference −31.17, SE −26.75, *p =* 0.182				
	Proteoglycans	Mean 7.67%, SE 2.22	Mean 13.52%, SE 3.77	Mean change 76.27%, SE 70.10, *p =* 0.198				
Hwang et al. (12)	Cell proliferation	NP	NP	NP	NP	NP	NP	The increased amount of HAp in the composite hydrogel resulted in enhanced cell proliferation. Thus, the beneficial effect of HAp on the biological activity of the levan composite hydrogel for its application as a dermal filler was clearly observed in proportion to the amount of HAp in the *in vitro* experiment (*p* < 0.05)
	Anti-wrinkle effect/collagen	NP	NP	NP	NP	NP	NP	The wrinkle areas of levan hydrogel and composite hydrogel were dramatically decreased over 90%, compared to VD3 (positive control)
Fan et al. (4)	Egfr, Smad2, Smad 3, procollagen, elastin, fibrillin	NP	NP	NP	NP	NP	NP	• week 4: Expressions of Egfr, Smad 2, procollagen, elastin were higher in Radiesse group compared to control (*p* < 0.005); no difference in Smad 3 and fibrillin expression was found. •week 4: The procollagen levels were significantly higher in the HAc-micro-HAp group than in the Radiesse group (p < 0.005). The Smad2 and fibrillin levels of the HAc-micro-HAp group were significantly higher than those of the Radiesse group *p* < 0.05, whereas the Smad3 levels were significantly lower (*p* < 0.05). •week 12: no difference between groups was found in any of the proteins
	Elastic fibers, vimestin, tropoelastin,	NP	NP	NP	NP	NP	NP	• Elastic fiber formation and tropoelastin were higher in Radiesse group compared to control (*p* < 0.05); no difference was found in vimentin in week 1 and 4, but higher levels in Radiesse group at week 8 and 12. •Hac-microHAp showed higher levels of Vimentin at week 1, 4 and 8 but not at week 12 compared to Radiesse, while only higher levels of Tropoelastin at week 12. •No difference in elastin fiber was observed except for week 1.
	Western blotting analysis	NP	NP	NP	NP	NP	NP	• Compared to control, Radiesse group showed higher levels of EGFR and Smad 7 (p < 0.05), but no difference in TGF-beta, P-MAPK ½, Smad 2/3 and Collagen 1.
Rovatti et al. (6)	Collagen morphology and vessel density	NP	NP	At T0, poorly refractive and thick collagen fibers were observed, whereas highly refractive and thin collagen fibers arranged in a net were detected at T4. Dynamic-optical coherence tomography enabled the visualization of increased vessel density	NA	NA	NA	NA
Yutskovskaya et al. (13)	Neocollagenesis: collagen type I expression	4.29 ± 0.72	• 4 months: 4.95 ± 1.22•7 months: 6.0 ± 0.0	• 4 months: increased, *p =* 0.04 •7 months: increased, *p* < 0.0001	NA	NA	NA	NA
	Neocollagenesis: collagen type III expression	2.38 ± 0.81	• 4 months: 5.26 ± 1.20•7 months: 2.59 ± 0.94	• 4 months: increased, *p* < 0.0001) •7 months, no significant difference	NA	NA	NA	NA
	Neo-elastogenesis: elastin expression	2.24	• 4 months: 2.95•7 months: 3.88	• 4 months: increased, *p* < 0.05 •7 months: increased, *p* < 0.0001				
	Neo angiogenesis: CD34	3.62	• 4 months: 4.63•7 months: 5.53	• 4 month: increased, *p* < 0.01 •7 month: increased, *p* < 0.0001	NA	NA	NA	NA
Coleman et al. (14)	Neocollagenesis, collagen	NP	NP	Increase: After injection of CaHA, increased collagen was seen over the time interval of 4 to 78 weeks intradermal but not subdermal	NA	NA	NA	NA
Yutskovskaya et al. (15)	Neocollagenesis, collagen type I	NP	• 4 months, mean staining intensity: 4.0 ± 1.44•9 month: 6.58 ± 1.1	NP	NP	• 4 months, mean staining intensity: 3.65 ± 1.65 •9 month: 4.8 ± 1.86	NP	• 4 months: Increased, collagen type I formation was found to be higher with CaHA than with HA gel, albeit non-significant (*p =* 0.0679). •9 months: increased, mean staining intensity for collagen type I was significantly greater after treatment with CaHA gel matrix than with HA gel (*p =* 0.0135)
	Neocollagenesis, collagen type III	NP	• 4 months: 5.2 ± 1.67•9 months: 3.7 ± 1.09	NP	NP	• 4 months: 4.2 ± 1.44 •9 months: 6.02 ± 0.82	NP	• 4 months: increased; the mean staining intensity of collagen type III was significantly greater with CaHA gel matrix than HA gel (*p =* 0.0052) •9 months: lower; The staining intensity for collagen type III was significantly lower at month 9 after treatment with CaHA gel matrix compared with HA gel (*p =* 0.0019)
	Elastin	NP	4 months: 2.8 ± 2.3 9 months: 5.2 ± 1.22	NP	NP	• 4 months: 1.0 ± 1.15 •9 months: 4.33 ± 1.27	NP	• 4 months: higher with CaHA (*p =* 0.0004) •9 months: higher with CaHA, (*p =* 0.0186)
	Ki-67	NP	• 4 months: 3.4% ± 2.08%•9 months: 6.2% ± 2.2%	NP	NP	• 4 months: 3.3% ± 2.4% •9 months: 4.5% ± 1.79%	NP	• 4 months: similar between groups (*p =* 0.2013) •9 months: higher with CaHA (*p =* 0.0011)
	Angiogenesis, H & Estained sections	NP	NP	NP	NP	NP	NP	More angiogenesis at both 4 and 9 months in CaHA group (*p* < 0.0002 and *p* < 0.0001, respectively)
	Inflammation, Grade of lymphohistiocytic infiltration	NP	NP	NP	NP	NP	NP	Less inflammation at both 4 and 9 month (*p =* 0.0108 and *p* < 0.0001, respectively)
	Mucoid edema	NP	NP	NP	NP	NP	NP	Month 4: Grade of mucoid edema was significantly lower with CaHA gel matrix compared with HA gel (*p* < 0.0001). Month 9: the grades were negligible for both treatments
Courderot-Masuyer et al. (3)	Contractile forces	NP	NP	Wrinkle and normal fibroblasts treated with CaHA developed higher contractile forces in comparison with non-treated fibroblast, *p* < 0.001	NP	NP	The forces developed by wrinkle fibroblasts in the presence of 2.5 ng/mL of TGFb1 were significantly increased compared to the control group (wrinkle fibroblasts), *p* < 0.001	NP
Figueredo et al. (16)	Total collagen density	• TT: 68 ± 4 •SCT: 67 ± 5	• TT: 72 ± 7•SCT: 74 ± 6	• TT: *p =* 0.05 •SCT: *p* < 0.01	NA	NA	NA	NA
	Neocollagenesis, collagen type I	• TT: 65 ± 11 •SCT: 64 ± 12	• TT: 64 ± 12•SCT: 67 ± 14	• TT: *p =* 0.17 •SCT: p =0.53	NA	NA	NA	NA
	Neocollagenesis, collagen type III	• TT: 56 ± 12 •SCT: 57 ± 11	• TT: 63 ± 9•SCT: 64 ± 10	• TT: *p =* 0.05 •SCT: *p =* 0.03	NA	NA	NA	NA
van Rozelaar et al. (7)	Collagen formation		82.6%	NP	96.2%		NP	No significant differences, *p =* 0.17
	Inflammation		4.9%	NP	2.4%		NP	No significant differences, *p >* 0.05

* Median (minimum, maximum). CaHA, calcium hydroxylapatite; PCL, polycaprolactone; SE, standard error; NP, not provided; NA, not applicable; SCT, superficial lamina with cannula technique; TT, tenting technique.

### Methodological rigor

We assessed the methodological rigor of the 12 included studies; for one of the studies, we used two checklists since it featured both an *in-vitro* and an animal experimental design. Detailed information on risk of bias assessment in the included studies is provided in Appendix 6. Of the three *in vitro* studies, two had a medium risk of bias and one of the studies a low risk of bias. The limitations of these studies involved incomplete information by the assessors of outcomes and unclear explanation of sample size calculation. Sometimes the statistical analysis and presentation of results were sub-optimal. The other domains showed low risk of bias. All animal studies suffered from high risk of bias in at least one domain and did not provide adequate information for assessment in almost half of the domains. The studies in general showed a high risk of bias during the processes of sequence generation, allocation concealment and did not provide information on blinding of intervention and outcome. The allocation concealment domain was considered at highest risk of bias, followed by the housing procedure which was not described in detail in most of the studies included and was thus difficult to assess. The studies were also performed usually in a very small sample size and lacked details in the other aspects, making other sources of bias difficult to analyze. Three out of four non-randomized studies were judged to be at serious risk of bias in at least one domain, but there was no critical risk of bias in any domain. The domain bearing the highest risk of bias was the measurement of outcomes due to flaws in the methodology and the blinding of assessors. The only randomized trial also showed insufficient information for proper assessment when it came to the risk of bias due to deviations from intended interventions and measurement of outcomes, having to do with the blinding of interveners and assessors.

### Summary of evidence

#### Cell proliferation

Different markers of cell proliferation were used across studies, including cell count, adenosine triphosphate (ATP) concentration, and Ki-67 marker. Using cell count as a measure of cell proliferation in a rat model, Yanatma et al. (11) showed no changes in number of fibroblast nuclei at either 2^nd^ or 4^th^ month in the CaHA group; compared to CaHA, polycaprolactone resulted in higher number of fibroblast nuclei at 4^th^ month but not at the 2nd month. Wollina et al. (10) used human HaCaT keratinocytes and human dermal fibroblasts to investigate effects of Radiesse in cell proliferation. Compared to negative controls, there was no observed change in cell proliferation and cell viability by CaHA based on cell count. Nevertheless, CaHA significantly increased ATP concentration in human dermal fibroblast cultures without changes in lactate dehydrogenase (LDH), a marker of cytotoxicity. Hwang et al. (12) showed cell proliferation increased proportionate with increases in the amount of CaHA in an *in vitro* experiment using human adult dermal fibroblasts. Yutskovskaya, et al. (15) in a randomized, split-face, histomorphologic study comparing CaHA with a hyaluronic acid-based dermal filler showed no difference between groups in cell proliferation (based on Ki-67 marker) at 4 months, but cell proliferation was higher in the CaHA group at 9 months.

#### Neocollagenesis

Hwang et al. (12) showed that collagen production increased in proportion to the amount of CaHA in an *in vitro* experiment of human adult dermal fibroblasts. Coleman et al. (14) in a single-arm experimental animal study reported that intradermal Radiesse resulted in increased collagen over 4 to 78 weeks but not when injected subdermal. Fan et al. (4) reported that Radiesse, compared to a control group, resulted in high gene expression of procollagen at week 4 in a mouse model; a hyaluronic acid-micro hydroxyapatite filler showed the highest levels. In contrast, no differences were observed between groups at week 12. In a 3-arm experimental study by Yanatma et al. (11) CaHA, compared with the control group, resulted in higher type I and III collagen H scored at both 2^nd^ or 4^th^ month; no differences were observed between CaHA and polycaprolactone. In a randomized clinical study, Yutskovskaya et al. (15) showed that Radiesse, compared to a hyaluronic acid-based dermal filler resulted in higher levels of collagen type III at month 4 but no significant difference in collagen type I. At month 9, the Radiesse group showed higher levels of collagen type I but lower levels of collagen type III. In a pre-post retrospective study, Rovatti et al. (6) reported that while at baseline poorly refractive and thick collagen fibers were observed, highly refractive, thin collagen fibers arranged in a net were observed at month 4 after injection of CaHA in the face. Another pre-post study by Yutskovskaya et al. (13) showed an increase in collagen type I and III expression 4 months after Radiesse injection in the neck and periocular area, and maintained for type I collagen at the month 7. Findings of an interventional study done by Figueredo et al. showed that mean total collagen density was increased in deep dermis by 9.5% from baseline value to 24 weeks after Radiesse injection. They illustrated that collagen Type III was increased after injection (*P* < 0.05) while the density of collagen Type I showed no significant change. In this study, investigators compared two injection techniques using Radiesse (16). In a Dutch prospective study with 1 year follow-up and based on magnetic resonance imaging, it was shown that injection of both poly-L-lactic acid and CaHA in the facial sites lead to collagen formation in more than 80% of HIV-positive patients during the 12 months period, with no significant differences between the groups (7).

#### Elastic fiber and elastin

Fan et al. (4) using a mouse model, showed higher gene expression of elastin at week 4 with Radiesse compared to control, while no difference was found at week 12. At all-time points (week 1, 4, 8, and 12), percentage of elastic fiber formation was higher in Radiesse compared to control. No difference in elastin gene expression or elastic fiber formation was found between Radiesse and hyaluronic acid-micro hydroxyapatite filler. Gonzalez et al. (5) evaluated formation of elastic fibers and elastin at baseline and 6 months after CaHA injection in 15 women in the sun-exposed right infra-auricular area with no comparison group, and showed increased levels only in some women. On average, no significant change was observed in the overall group. Yutskovskaya (13) in a pre-post study reported an increase in elastin formation at both 4 and 9 months after Radiesse injection in the neck and periocular area. In an earlier randomized study, Yutskovskaya (15) showed that Radiesse, compared to a hyaluronic acid-based dermal filler, lead to higher elastin at month 4, as well as at month 9.

#### Angiogenesis

Angiogenesis was evaluated by different approaches, including assessment of vessel density, counting the number of capillary-type vessels and by assessing CD-34 markers. Two single-arm observational studies (6, 13) showed an increase in angiogenesis after CaHA injection. Similar findings were obtained in a randomized clinical study (15) showing increased angiogenesis at both 4 and 9 months with Radiesse compared to hyaluronic acid-based dermal filler.

#### Other skin regeneration mechanisms

Five studies assessed other aspects of skin regeneration. Courderot-Masuyer et al. (3) in an *in vitr*o study using wrinkle fibroblasts and normal aged fibroblasts from facelifts of women, showed that wrinkle and normal fibroblasts treated with Radiesse developed higher contractile forces in comparison with non-treated fibroblasts; the difference between comparison groups was not provided. Fan et al. (4) using a mouse model, showed that at week 4, gene expressions of epidermal growth factor receptor (EGFR, a cell surface protein that binds to epidermal growth factor) and Suppressor of Mothers against Decapentaplegi-2 (smad 2) were higher in the Radiesse group compared to control; no difference in Smad 3 and fibrillin expression was found. SMAD are a family of structurally similar proteins that are the main signal transducers for receptors of the transforming growth factor beta (TGF-B) superfamily. At week 12, no differences were found between groups. When comparing Radiesse with a hyaluronic acid-micro hydroxyapatite filler, the latter showed only a higher gene expression of Smad 2 and fibrillin at week 4, while no differences in other genes or at week 12 were observed. Similarly, Vimentin (a major constituent of the intermediate filament family of proteins) and Tropoelastin (the soluble precursor of elastin) formation was higher in the Radiesse group as compared to control at most time points (week 1, 4, 8 and 12). In addition, Radiesse showed higher levels of EGFR and Smad 7, but no difference in TGF-β, P-MAPK ½, (mitogen-activated protein kinases), Smad 2, and Smad3. Gonzalez et al. (5) showed no change in proteoglycans between baseline and 6 months after CaHA injection in the sun-exposed right infra-auricular area of 15 women; no comparison group was reported. Yutskovskaya et al. (15) reported less inflammation and lower grade of mucoid edema after Radiesse injection compared to hyaluronic acid-based dermal filler. In line with these findings, in a 1-year follow-up Dutch prospective cohort of 82 HIV-patients, signs of inflammation were observed in two and one patients treated with CaHA and polpoly-L-lactic acid injections, respectively, with no significant differences between the two treatment groups (7).

## Discussion

Our systematic review shows several mechanisms through which CaHA can exert its skin regenerative effects, including cell proliferation, collagen and elastin synthesis, as well as stimulation of angiogenesis. Limited data also show anti-inflammatory effects of CaHA.

Cell proliferation is considered key process of tissue regeneration (17). Fibroblast migration and proliferation as well as synthesis of extracellular matrix constitute the hallmarks of the proliferative phase of the dermal repair process (17). While current evidence does not show consistent findings on fibroblast proliferation stimulated by CaHA as measured by cell count, our findings indicate that CaHA may affect cell proliferation as reflected by higher levels of Ki-67; this process was not accompanied by increased Lactic Acid Dehydrogenase (LDH) levels, an indicator of potential toxicity. These findings indicate CaHA may stimulate proliferation of collagen-producing cells, which could partially explain the increase in collagen production. While the role of Ki-67 is not fully understood, evidence suggests Ki-67 expression affects heterochromatin organization in proliferating cells, thereof controlling gene expression (18). In addition, our results show that CaHA injection was associated with increased expression of Epidermal Growth Factor Receptor (EGFR) and Smad2 genes, which are involved in cell proliferation. The stimulation of gene expression by CaHA may not last long, considering that gene expression of EGFR and Smad 2 was observed at 4week but not at the 12 weeks.

Our findings on stimulation of collagen synthesis by CaHA are in line with other data which were not included in our present summary due to not fulfilling our inclusion criteria (19, 20). While data are not consistent, in general evidence suggests CaHA may induce synthesis of collagen type I and III in early phases, and then gradually collagen type I replacing collagen type III, consistent with the natural process of remodeling. Collagen type I constitutes 80–85% of the dermal extracellular matrix in the skin, while collagen type III constitutes about 8–11% (21). Both these collagen types have an estimated half-life of around 15 years in the skin, indicating the turnover is very slow (21). Collagen production decreases with age, and elderly people show up to 75% less production in collagen compared to younger age groups (22, 23). Reduction of type I and III collagen is a characteristic feature of a photodamaged, and chronologically aged skin characterized by morphological and mechanical changes resulting in wrinkle formation, loss of elasticity and dryness (24). CaHA therefore can counteract these processes by inducing collagen synthesis, and persistent collagen type I synthesis can provide strength and resilience to skin (25). Future research is needed to explore different time-intervals of collagen synthesis and number of CaHA injections needed for optimal results.

Elastin, the main component of elastic fibers, was shown to increase after CaHA injection. Elastic fiber production provides stretch, recoil, and elasticity to the skin, and is integral for a youthful appearance (26). Age and exposure to environmental factors such as sun, trigger degradation of elastic fibers, limiting wound healing capacity and enhancing the appearance of scars and stretch marks in the skin (26). Evidence shows that CaHA fillers have the potential to regenerate elastic fibers by stimulating elastin production, which could also be a potential mechanism through which CaHA fillers improve skin appearance and texture. Nevertheless, studies in animal models suggest that the effect of CaHA fillers is time-dependent, and thus more research is needed to understand how long CaHA‘s stimulatory effect on elastin lasts.

Treatment with CaHA also stimulated angiogenesis which may indicate improved blood flow and improved nutrient delivery to the skin. This is in line with the lower grade of inflammation and mucoid edema observed after CaHA injection, both important to regenerative and wound healing processes. The cellular mechanisms involved in angiogenesis and lower inflammation induced by CaHA remain to be elucidated.

To the best of our knowledge, our study is the first comprehensive systematic review of mechanisms that could explain skin-regenerative effects of CaHA. Previous work on this topic has been narrative or expert reviews, which are not systematic and are more prone to bias. Nevertheless, several limitations remain in the present study. First, some studies did not show baseline values of the outcomes they assessed, challenging the interpretation of their findings. While differences in outcomes can be observed at the end of study, the difference depends also on the baseline values. Second, for some studies no comparison group was available, and thus it is not clear whether the observed effects are due to CaHA or other factors, and if an effect exists, to which extent CaHA makes a difference. Third, different follow-up times were used across studies, precluding our ability to explore time-dependent effects of CaHA. Lastly, given the methodological concerns in the included studies, our findings should be interpreted with caution.

Overall, evidence indicates several mechanisms through which CaHA may exert its skin regenerative effects. Future higher quality clinical studies with larger sample size and better statistical approaches, are needed to better understand the CaHA mechanisms of action such as cell proliferation, collagen synthesis, and angiogenesis.

## Data availability statement

The original contributions presented in the study are included in the article/Supplementary material, further inquiries can be directed to the corresponding author.

## Author contributions

TM and HD conceptualized the research and supervised the project implementation. MA, RM, HD, and TM were involved in the systematic review design, protocol writing, study selection procedure, and data interpretation. CN, HD, TP, and JK designed the search strategy. CN performed the literature search. MA, RM, and TM were involved in screening abstracts and full articles, data extraction and quality assessment of included studies, and drafting the manuscript. HD, TP, and JK reviewed and edited the draft. All authors have approved the final version of this manuscript.
