# Supplementary material for: Skin regeneration-related mechanisms of Calcium Hydroxylapatite (CaHA): a systematic review
Source: Front Med (Lausanne). 2023 Jun 2;10:1195934. doi: 10.3389/fmed.2023.1195934 (PMC10273839; doi:10.3389/fmed.2023.1195934)
Supplement: Supplementary file 1 [file Table_1.docx]

Supplementary Material

Appendix 1. PRISMA checklist

Appendix 2. Search Strategy

Appendix 3. Checklists used to assess risk of bias

Appendix 4. Characteristics of intervention and control used within each study

Appendix 5. Outcomes, and statistical methods

Appendix 6. Methodological Rigor in the included studies

Appendix 1. PRISMA checklist

| **Section and Topic** | **Item #** | **Checklist item** | **Location where item is reported** |
| --- | --- | --- | --- |
| **TITLE** | | |  |
| Title | 1 | Identify the report as a systematic review. | Page 1 |
| **ABSTRACT** | | |  |
| Abstract | 2 | See the PRISMA 2020 for Abstracts checklist. | Page 2 |
| **INTRODUCTION** | | |  |
| Rationale | 3 | Describe the rationale for the review in the context of existing knowledge. | Page 3 |
| Objectives | 4 | Provide an explicit statement of the objective(s) or question(s) the review addresses. | Page 3 |
| **METHODS** | | |  |
| Eligibility criteria | 5 | Specify the inclusion and exclusion criteria for the review and how studies were grouped for the syntheses. | Page 4 |
| Information sources | 6 | Specify all databases, registers, websites, organisations, reference lists and other sources searched or consulted to identify studies. Specify the date when each source was last searched or consulted. | Page 3-4 |
| Search strategy | 7 | Present the full search strategies for all databases, registers and websites, including any filters and limits used. | Page 3-4 |
| Selection process | 8 | Specify the methods used to decide whether a study met the inclusion criteria of the review, including how many reviewers screened each record and each report retrieved, whether they worked independently, and if applicable, details of automation tools used in the process. | Page 4 |
| Data collection process | 9 | Specify the methods used to collect data from reports, including how many reviewers collected data from each report, whether they worked independently, any processes for obtaining or confirming data from study investigators, and if applicable, details of automation tools used in the process. | Page 4-5 |
| Data items | 10a | List and define all outcomes for which data were sought. Specify whether all results that were compatible with each outcome domain in each study were sought (e.g. for all measures, time points, analyses), and if not, the methods used to decide which results to collect. | Page 4-5 |
|  | 10b | List and define all other variables for which data were sought (e.g. participant and intervention characteristics, funding sources). Describe any assumptions made about any missing or unclear information. | Page 4-5 |
| Study risk of bias assessment | 11 | Specify the methods used to assess risk of bias in the included studies, including details of the tool(s) used, how many reviewers assessed each study and whether they worked independently, and if applicable, details of automation tools used in the process. | Page 4 |
| Effect measures | 12 | Specify for each outcome the effect measure(s) (e.g. risk ratio, mean difference) used in the synthesis or presentation of results. | Page 4-5 |
| Synthesis methods | 13a | Describe the processes used to decide which studies were eligible for each synthesis (e.g. tabulating the study intervention characteristics and comparing against the planned groups for each synthesis (item #5)). | Page 5 |
|  | 13b | Describe any methods required to prepare the data for presentation or synthesis, such as handling of missing summary statistics, or data conversions. | Page 5 |
|  | 13c | Describe any methods used to tabulate or visually display results of individual studies and syntheses. | Page 5 |
|  | 13d | Describe any methods used to synthesize results and provide a rationale for the choice(s). If meta-analysis was performed, describe the model(s), method(s) to identify the presence and extent of statistical heterogeneity, and software package(s) used. | Page 5 |
|  | 13e | Describe any methods used to explore possible causes of heterogeneity among study results (e.g. subgroup analysis, meta-regression). | Page 5 |
|  | 13f | Describe any sensitivity analyses conducted to assess robustness of the synthesized results. | Page 5 |
| Reporting bias assessment | 14 | Describe any methods used to assess risk of bias due to missing results in a synthesis (arising from reporting biases). | Page 5 |
| Certainty assessment | 15 | Describe any methods used to assess certainty (or confidence) in the body of evidence for an outcome. | Page 5 |
| **RESULTS** | | |  |
| Study selection | 16a | Describe the results of the search and selection process, from the number of records identified in the search to the number of studies included in the review, ideally using a flow diagram. | Page 5 |
|  | 16b | Cite studies that might appear to meet the inclusion criteria, but which were excluded, and explain why they were excluded. | Flow chart |
| Study characteristics | 17 | Cite each included study and present its characteristics. | Page 5 |
| Risk of bias in studies | 18 | Present assessments of risk of bias for each included study. | Page 6 |
| Results of individual studies | 19 | For all outcomes, present, for each study: (a) summary statistics for each group (where appropriate) and (b) an effect estimate and its precision (e.g. confidence/credible interval), ideally using structured tables or plots. | Page 5-9 |
| Results of syntheses | 20a | For each synthesis, briefly summarise the characteristics and risk of bias among contributing studies. | Page 5-9 |
|  | 20b | Present results of all statistical syntheses conducted. If meta-analysis was done, present for each the summary estimate and its precision (e.g. confidence/credible interval) and measures of statistical heterogeneity. If comparing groups, describe the direction of the effect. | NA |
|  | 20c | Present results of all investigations of possible causes of heterogeneity among study results. | Page 5-9 |
|  | 20d | Present results of all sensitivity analyses conducted to assess the robustness of the synthesized results. | NA |
| Reporting biases | 21 | Present assessments of risk of bias due to missing results (arising from reporting biases) for each synthesis assessed. | Page 5-6 |
| Certainty of evidence | 22 | Present assessments of certainty (or confidence) in the body of evidence for each outcome assessed. | NA |
| **DISCUSSION** | | |  |
| Discussion | 23a | Provide a general interpretation of the results in the context of other evidence. | Page 9 |
|  | 23b | Discuss any limitations of the evidence included in the review. | Page 11 |
|  | 23c | Discuss any limitations of the review processes used. | Page 11 |
|  | 23d | Discuss implications of the results for practice, policy, and future research. | Page 9-11 |
| **OTHER INFORMATION** | | |  |
| Registration and protocol | 24a | Provide registration information for the review, including register name and registration number, or state that the review was not registered. | Page 3 |
|  | 24b | Indicate where the review protocol can be accessed, or state that a protocol was not prepared. | Page 3 |
|  | 24c | Describe and explain any amendments to information provided at registration or in the protocol. | NA |
| Support | 25 | Describe sources of financial or non-financial support for the review, and the role of the funders or sponsors in the review. | Page 11 |
| Competing interests | 26 | Declare any competing interests of review authors. | Page 11 |
| Availability of data, code and other materials | 27 | Report which of the following are publicly available and where they can be found: template data collection forms; data extracted from included studies; data used for all analyses; analytic code; any other materials used in the review. | All data are available in main text and supplemental material. |

Appendix 2. Seach strategy

Embase

((radiesse OR CaHA OR calcium-hydroxyapatit* OR calcium-hydroxylapatit*):ab,ti,kw) NOT ([Conference Abstract]/lim OR 'letter'/de OR 'note'/de OR 'review'/exp OR (systematic-review* OR meta-analys* OR metaanalys*):ti)

Medline

((radiesse OR CaHA OR calcium-hydroxyapatit* OR calcium-hydroxylapatit*).ab,ti,kf.) NOT ((news OR congres* OR abstract* OR book* OR chapter* OR dissertation abstract*).pt. OR (systematic-review* OR meta-analys* OR metaanalys*).ti.)

Web of Science

TS=((radiesse OR CaHA OR calcium-hydroxyapatit* OR calcium-hydroxylapatit*)) NOT TI=((systematic-review* OR meta-analys* OR metaanalys*)) AND DT=(Article OR Review OR Letter OR Early Access)

Cochrane

((radiesse OR CaHA OR calcium-hydroxyapatit* OR calcium-hydroxylapatit*):ab,ti,kw) NOT ((systematic-review* OR meta-analys* OR metaanalys*):ti)

Google Scholar

radiesse|CaHA|"calcium hydroxyapatite"|"calcium hydroxylapatite" -orbital|patulous|Eustachian|vocal|dental|teeth|tooth|voice|osteoporose| osteomyelitis|bone|scars

Appendix 3. Checklists used to assess risk of bias

| **Study Design** | **Checklist** | **Link to checklist** |
| --- | --- | --- |
| In vitro | QUIN | https://pubmed.ncbi.nlm.nih.gov/35752496/ |
| In vivo/ animal experimental | Systematic Review Centre for Laboratory Animal Experimentation Risk of Bias tool. | https://pubmed.ncbi.nlm.nih.gov/24667063/ |
| Non-randomized human studies | ROBINS-I – Risk Of Bias In Non-Randomized Studies of Interventions | https://pubmed.ncbi.nlm.nih.gov/27733354/ |
| Randomized Clinical Trials | Cochrane Collaboration’s Tool Risk of Bias 2 (RoB2) | https://pubmed.ncbi.nlm.nih.gov/31462531/ |

Appendix 4. Characteristics of intervention and control used within each study

| **Author, year** | **Injected area/tissue** | **Intervention** | | | | | **Control** | | | | |
| --- | --- | --- | --- | --- | --- | --- | --- | --- | --- | --- | --- |
|  |  | **n** | **Brand** | **Dosage** | **Dilution** | **Injection tool** | **n** | **Brand** | **Dosage** | **Dilution** | **Injection tool** |
| Wollina U, 2018 | Human HaCaT keratinocytes  and human dermal fibroblasts | NP | Radiesse 3.0 cc  (Merz Pharmaceutical GmbH, Frankfurt/ Main, Germany) | NP | NP | NP | NP | HaCaT keratinocytes or  human dermal fibroblasts grown in DMEM served as a negative control. Triton-X100 1% was used as a positive control for cytotoxicity. | NP | NP | NP |
| Yanatma I, 2020 | An area of 4 × 4 cm2  was shaved in the back of the subjects. Two points at a distance of  12 cm from the tip of the nose and 1.5 cm bilaterally from the spinal  midline was marked for application. | 10 | NS | 0.2 mL | NS | 25-guage needle | Group 1, 10; Group 2, 10 | Group 1, Polycaprolactone (PCL); Group 2, no intervention | 0.2 mL | NS | 25-guage needle |
|  |  |  |  |  |  |  |  |  |  |  |  |
| Gonzalez N, 2019 | Sun-exposed right infra-auricular area | 15 | NS | NS | NS | NS | NA | NA | NA | NA | NA |
| Hwang Y, 2021 | Human adult dermal fibroblast and dorsal skin of the mouse | Cell experiment, n=4 | HAp Radiesse | Cell experiment; 0.1 to 5 wt%  Animal experiment; the hydrogel (300 mL, sol-state,  prepared at 4 C) | Cell experiment; Dissolved in deionized  water for 24 h using a rotary shaker at 4 C | For animal experiment, 1 mL syringe (24G). | NA | NA | NA | NA | NA |
| Fan Y, 2019 | The panniculus adiposus layer and the panniculus carnosus of the back skin. | 20 | HAp (Radiesse, Raleigh, North Carolina) | 200 μl | NS | a 30-guage needle | 4* | NA | NA | NA | NA |
| Rovatti PP, 2020 | Right side of the mid-lower face and  the left side. | 40 | Hyperdiluted CaHA 1:2 technique, no brand provided | 6 mL | Each  1.5 mL CaHA syringe was diluted with 0.5 mL of 1%  lidocaine and 3mL of 0.9% saline | 5 G 50 mm cannula | NA | NA | NA | NA | NA |
| Yutskovskaya YA, 2017 | Beginning laterally and moving toward the medial line of the neck and decolletage, as well as in periauricular area | 20 | CaHA, MERZ | The total volume of the dilutes product was at the discretion of the investigator, depending on skin thickness | Each 1.5 mL syringe was diluted with preserved saline 0.9% to one of the three dilutions: 1:2, 1:4 or 1:6 | A 27-gauge, 19 mm needle | NA | NA | NA | NA | NA |
| Coleman KM, 2008 | Lateral to the lumbar spine | 6 | CaHA, Radiesse | 0.2 to 0.3 cm^3^ | NS | NS | NA | NA | NA | NA | NA |
| Yutskovskaya Y et al. 2014 | Postauricular area on the left  side. | 24 | CaHA, Radiesse | 0.1mL | NS | Supraperiostal administration | 24 | HA gel | 0.1mL | NS | Supraperiostal administration |
| Courderot-Masuyer, C, 2016 | Facelifts (operative  waste) | 3 | CaHA, Radiesse | 3 mL | Dissolved in 27 mL of  DMEMc. | Syringe | 3 | TGFb1 | 2.5 ng/mL | NS | NS |
| Figueredo, 2020 | Skin biopsies (3-mm punch) from the dorsum of the hands | 13 (26 hands) | CaHA, Radiesse | 3 mL | NA | 1- tenting technique: 27-G needle  2- SCT, superficial lamina technique: 25-G cannula | NA | NA | NA | NA | NA |
| Rozelaar L, 2014 | One or more facial sites in a fan-like manner | 41 | CaHA, brand not specified | 0.5 mL | pretreatment with 0.5 mL of lidocaine 2% thoroughly  mixed | 27-gauge, 1.75-inch needle | 41 | Poly-L-lactic acid ( | NS | 5 mL of sterile H2O and 2 mL of  lidocaine 1%. | 26-gauge, 1-inch needle |

NS, not specified
*There were three other groups, injected with 200 μl of HAc (Restylane, Upp- sala, Sweden), HAc-nano-HAp, or HAc-micro-HAp

**Appendix 5.** Outcomes, and statistical methods

| **Author, year** | **Outcome** | **Method** | **Adjustment** | **Measure of association, test** |
| --- | --- | --- | --- | --- |
| Wollina U, 2018 | Cell proliferation | Luminometric ad-enosine triphosphate (ATP), TP-LiteTM-M Assays (PerkinElmer LAS (Germany) GmbH, Rodgau, Germany). | No | mean±standard deviation (SD), Students T-test |
|  | Cytotoxicity | Liberated lactate dehydrogenase (LDH), Calorimetric Cytotoxicity  Detection Kit (Roche Diagnos-tics). |  |  |
| Yanatma I, 2020 | Total collagen Density | Percent of collagen areas in all images of all groups were  calculated by using the ImageJ program (National Institutes of  Health, Bethesda, Maryland). | No | Median, minimum, and maximum  values. Kruskal-Wallis test and then Conover pairwise comparison method were used for independent group comparisons. The time-variance of the groups was examined using the Wilcoxon test. |
|  | Dermis thickness and cell count | It was determined by calculating  the average thickness of at least three different points in  the cross-sections. The mean numbers of fibroblasts were  found by counting 10 different areas at ×40 objective in each section. |  |  |
|  | Type 1 and 3 collagen immunoreactivity levels of fibroblasts | H-score (H-score = staining intensity × percentage of stained  cells) based on the staining intensity (no staining = 0, weak  staining = 1, medium intensity staining = 2, strong intense staining = 3)  and percentage of stained cells |  |  |
| Gonzalez N, 2019 | Elastic fibers, Elastin, Proteoglycan | Detection of elastin by immunohistochemistry (IHC)  was performed using a rabbit polyclonal antibody directed against elastin samples pooled from pig, human, dog, rat, chicken, and cow (Abcam, ab21610). The other 2 sections were stained with  alcian blue pH 2.5. All stained slides were then  scanned at 20· magnification (Aperio ScanScope XT, Thornwood, NY). The stained slides and copies of the digital images were sent for pathology and image analysis. The image analysis was performed as follows: between 3 and 4 images were photographed of  each biopsy with a 2 light microscope using a 20·  objective (Zeiss Axioplan, Thornwood, NY).  Computer-assisted image analysis was accomplished  using the customized software (Zeiss AxioVision,  Thornwood, NY) that enables the user to select a specific color (e.g., brown for elastin, royal blue for  Alcian blue). A range for the color intensity of a given stain (e.g., light to dark brown for elastin) is set through a histogram, and this setting was not changed during the entire analysis. The software calculates the area of the color in pixels and the total area of the dermis. Dividing the total area into the area occupied by the color is the percentage of the  stained component. | No | Mean percent change, Students T-test |
| Hwang Y, 2021 | Cell proliferation,  Wrinkle effect (collagenesis) | The cell proliferation of the composite hydrogels were measured using CCK-8 assay.  To quantify the amount of collagen production in the  skin, the skin samples containing the injection sites were collected and a hydroxyproline assay was conducted [32]. The skin biopsies around the injection site (1 mm2) were obtained at each time point (1, 4, and 8 weeks). Next, 6 N HCl was applied to the skin biopsy in polypropylene cryogenic vials (Corning, NY, USA) and incubated  at 80 C for 1 day to hydrolyze the skin. Then, the samples were neutralized at 80 C in a vacuum oven with NaOH till they were dried, and then the amount of hydroxyproline was analyzed at 550 nm with Ehrlich’s reagent | No | All analyses in this study were conducted at least four times. A  Student’s t-test and analysis of variance (ANOVA) were used for  statistical analysis |
| Fan Y, 2019 | Gene and protein expression | - Real-time PCR was performed in an ABI 7500 (Applied Biosystems, Inc., Foster City, CA, USA) with SYBR Premix Ex Taq TM (TaKaRa, Otsu, Japan) as follows: 15 min at 95 °C, followed by 40 cycles of 10 s at 95 °C, 15 s at 60 °C, and 30 s at 72 °C. The average threshold cycle for each gene was determined from triplicate reactions - Western blot analysis - Histological analysis: The sections were stained with Verhoeff–Van Gieson to visualize the pro- duction of elastin. Representative images were analyzed using image analysis software (ImageJ; National Institutes of Health, Bethesda, MD, USA) (Leica QWin V3; Leica Mi- crosystems Cambridge, England, UK). | No | Kruskal–Wallis test, with IBM SPSS software version 20.0 (Armonk, NY, USA). Step-up Mann–Whitney tests were performed with a multiple com- parison adjustment |
| Rovatti PP, 2020 | Collagen morphology and Vessels density | Reflectance confocal microscopy (RCM) and dynamic optical  coherence tomography (D-OCT) images acquired on the right cheek (5 mm below the zygomatic arch), following a standard protocol | No | Students T-test |
| Yutskovskaya YA, 2017 | Collagen I and II expression; Elastin and angiogenesis (CD 34) | Monoclonal antibodies against collagen type I (Sigma-Aldrich Corporation, St. Louis, MO; 1:4000 dilution), collagen type III (Novocastra Leica Biosystems, Newcastle upon Tyne, UK; 1:200 dilution), and CD34 (DAKO, Glostrup, Denmark) were used. | No | NS |
| Coleman KM, 2008 | Neocollagenesis | Collagen content was quantified using picrosirius red (PSR) staining under polarized light with photometric analysis | NS | NS |
| Yutskovskaya Y et al. 2014 | Collagen types I and III, elastin, and also Ki-67 and angiogenesis | Microphotography was performed using an Olympus BX41 microscope (Olympus America Inc., Melville, NY). Ultrasound scanning was performed at a frequency of 45 MHz using a SkinScanner DUB 22–75 MHz (taberna pro medicum GmbH, Lüneburg, Germany). Punch biopsies (96 biopsies from 24 participants) were fixed in 10% neutral formalin and embedded in paraffin according to standard protocols. Serial 4 μm paraffin sections were prepared and stained with hematoxylin-eosin (H&E. Tissue samples extracted through punch biopsies at months 4 and 9 after treatment were analyzed by both qualitative and quantitative measures for collagen types I and III and elastin expression. Qualitative and quantitative measures of Ki-67, lymphohistiocytic infiltration and angiogenesis were also taken at these timepoints. For immunohistochemistry, sections were stained after antigen unmasking in retriever solution, according to standard protocols. Monoclonal antibodies against collagen type I (Sigma- Aldrich Corporation, St. Louis, MO; used at 1:4000 dilution), collagen type III (Sigma Aldrich Corporation; used at 1:8000 dilution), elastin (Novacastra Leica Biosystems, Newcastle upon Tyne, UK; used at 1:200 dilution) and Ki-67 (RTU DaKo, Glostrup, Denmark; used at 1:100 dilution) were used. A semi-quantitative method was used to analyze the immunohistochemistry results, with 10 fields of vision studied at high magnification (x400) in two sections, according to the standard scale. Staining intensity was scored as weak (2 points), moderate (4 points), strong (6 points) or hyperexpression (8 points). Epidermal expression of Ki-67 was evaluated by the mean percentage of positively-stained cell nuclei among a sample of 300 epithelial cells. Angiogenesis was measured by counting the number of capillary-type vessels per 10 fields of vision in dermal tissue slides stained with H&E viewed at high magnification (x400). | No | Test of Wilcoxon |
| Courderot-Masuyer, C, 2016 | Contractile forces of  human wrinkle and normal aged fibroblasts | Fibroblasts were embedded three dimensionally in hydrated collagen gels using a modified modified version of the technique developed by Bell.11 Briefly, 2 mL of 1.76 9 concentrated Dulbecco’s modified Eagle’s medium (with penicillin/streptomycin, NaOH, and NaHCO3) was added to 1 mL of a solution of type I rat tail collagen (2 mg/mL, Jacques Boy,Reims, France) and to 0.33 mL of the suspension of living fibroblasts in the culture medium. The lattice mixture was poured into a rectangular culture well of the GlaSbox and polymerized in 30 min at 37°°C. Two  milliliters of culture medium were added. The GlaSbox  was then placed into a humidified incubator at 37°°C,  and force measurements were started immediately for 24 h. The results were expressed as contractile forces (arbitrary unit) according to the time allowing the quantification of contractile forces developed by living  fibroblasts in the presence or not of TGFb1 and CaHa | No | Data were expressed as mean ± sem. A variance analysis with two factors (group and  time) was carried out for the study of measurement of contractile forces, followed if necessary by a Fisher test |
| Figueredo, 2020 | Total collagen density,  Neocollagenesis, collagen type I,  Neocollagenesis, collagen type III | Skin biopsies (3-mm punch) from the dorsum of the hands were taken between the fourth and fifth metacarpal at T0 and T25. The specimens were fixed in 10% buffered formalin, embedded in paraffin, and processed for Picrosirius red and immunohistochemistry (DAB) staining for Collagen I (Abcam: clone ab34710) and III (Abcam: clone ab34710).  The slides were photographed (at ·100 magnification), focusing on the deep dermis, and the images were analyzed using ImageJ 1.46v software to quantify the density of collagen stain (as a percentile) and the intensity of immunohistochemistry staining, which ranged from 0 (no staining) to 255 (maximum staining). | No | quantitative data were reported as the mean (SD) or median (p25–p75) if normality was not confirmed by the Shapiro-Wilk test.19  The unit of analysis was each hand-nested within each participant, and the outcomes were compared according to time and to groups (over time) using linear mixed-effects models (post hoc Sidak). |
| Razelaar L, 2014 | Collagen formation  Inflammation | Collagen formation was defined as well-demarcated, pleomorphic,  hypointense subcutaneous tissue  Not specified how inflammation was assessed |  |  |

Appendix 6. Methodological Rigor in the included studies

| ROBINS-I – Risk Of Bias In Non-Randomized Studies  of Interventions |  |  |
| --- | --- | --- |
| **Rovatti PP, 2020** | | |
| Domain | Risk of bias | Comment |
| bias due to confounding | Low |  |
| bias in selection of participants into the study | Moderate | Start of follow-up and start of intervention, do not coincide for all participants |
| Bias in classification of intervention | Low |  |
| Bias due to deviations from intended interventions | Low |  |
| Bias due to missing data | Low |  |
| Bias in measurement of outcomes | Serious (favors experimental) | Subjective evaluation of outcomes |
| Bias in selection of the reported result | Low |  |
| **The study is judged to be at serious risk of bias in at least one domain, but not at critical risk of bias in any domain.** | | |
|  |  |  |
|  |  |  |
| **Yutskovskaya YA, 2017** | | |
| Domain | Risk of bias | Comment |
| bias due to confounding | Low |  |
| bias in selection of participants into the study | Low |  |
| Bias in classification of intervention | Low |  |
| Bias due to deviations from intended interventions | Low |  |
| Bias due to missing data | Low |  |
| Bias in measurement of outcomes | Serious | non blinded ultrasound assessment, subjective visual assessment |
| Bias in selection of the reported result | low |  |
| **The study is judged to be at serious risk of bias in at least one domain, but not at critical risk of bias in any domain.** | | |
| *author is sponsored by the manufacturing company and the study is sponsored by the manufacturing company | | |
|  |  |  |
| **van Rozelaar, 2014** | | |
| ***Domain*** | ***Risk of bias*** | ***Comment*** |
| bias due to confounding | Low |  |
| bias in selection of participants into the study | Low |  |
| Bias in classification of intervention | Moderate | Treatment with PLLA and CaHA was repeated as necessary every 1 to 2 months during the first 6 months. No clear definition on which patients received PLLA and which CaHA. |
| Bias due to deviations from intended interventions | Low |  |
| Bias due to missing data | Serious | 33 out of 82 patients lost to assessment follow up. |
| Bias in measurement of outcomes | Low |  |
| Bias in selection of the reported result | Low |  |
| **The study is judged to be at serious risk of bias in at least one domain, but not at critical risk of bias in any domain.** | | |
|  |  |  |
|  |  |  |
| **Figueredo, 2020** | | |
| ***Domain*** | ***Risk of bias*** | ***Comment*** |
| bias due to confounding | Low |  |
| bias in selection of participants into the study | Low |  |
| Bias in classification of intervention | Low |  |
| Bias due to deviations from intended interventions | Low |  |
| Bias due to missing data | Low |  |
| Bias in measurement of outcomes | Low |  |
| Bias in selection of the reported result | Low |  |
| **The study is judged to be at low risk of bias for all domains** | | |

| **ROBINS-I – Risk Of Bias In Non-Randomized Studies of Interventions** | | |
| --- | --- | --- |
| **Rovatti PP, 2020** | | |
| Domain | Risk of bias | Comment |
| bias due to confounding | Low |  |
| bias in selection of participants into the study | Moderate | Start of follow-up and start of intervention do not coincide for all participants |
| Bias in classification of intervention | Low |  |
| Bias due to deviations from intended interventions | Low |  |
| Bias due to missing data | Low |  |
| Bias in measurement of outcomes | Serious (favors experimental) | Subjective evaluation of outcomes |
| Bias in selection of the reported result | Low |  |
| **The study is judged to be at serious risk of bias in at least one domain, but not at critical risk of bias in any domain.** | | |
|  |  |  |
|  |  |  |
| **Yutskovskaya YA, 2017** | | |
| Domain | Risk of bias | Comment |
| bias due to confounding | Low |  |
| bias in selection of participants into the study | Low |  |
| Bias in classification of intervention | Low |  |
| Bias due to deviations from intended interventions | Low |  |
| Bias due to missing data | Low |  |
| Bias in measurement of outcomes | Serious | non blinded ultrasound assessment ; subjective visual assessment |
| Bias in selection of the reported result | low |  |
| **The study is judged to be at serious risk of bias in at least one domain, but not at critical risk of bias in any domain.** | | |
| *author and study sponsored by the manufacturing company | | |
|  |  |  |
| **van Rozelaar, 2014** | | |
| ***Domain*** | ***Risk of bias*** | ***Comment*** |
| bias due to confounding | Low |  |
| bias in selection of participants into the study | Low |  |
| Bias in classification of intervention | Moderate | Treatment with PLLA and CaHA was repeated as necessary every 1 to 2 months during the first 6 months. No clear definition on which patients received PLLA and which CaHA. |
| Bias due to deviations from intended interventions | Low |  |
| Bias due to missing data | Serious | 33 out of 82 patients lost to assessment follow up. |
| Bias in measurement of outcomes | Low |  |
| Bias in selection of the reported result | Low |  |
| **The study is judged to be at serious risk of bias in at least one domain, but not at critical risk of bias in any domain.** | | |
|  |  |  |
|  |  |  |
| **Figueredo, 2020** | | |
| ***Domain*** | ***Risk of bias*** | ***Comment*** |
| bias due to confounding | Low |  |
| bias in selection of participants into the study | Low |  |
| Bias in classification of intervention | Low |  |
| Bias due to deviations from intended interventions | Low |  |
| Bias due to missing data | Low |  |
| Bias in measurement of outcomes | Low |  |
| Bias in selection of the reported result | Low |  |
| **The study is judged to be at low risk of bias for all domains** | | |

| **Overall risk of bias according to RoB 2** | | |
| --- | --- | --- |
| **Yutskovskaya Y et al. 2014** | | |
| **Domain** | **Risk of bias** | **Comment** |
| Bias arising from the randomization process | Low |  |
| Bias due to deviations from intended interventions | NI | intervention was not blinded |
| Bias due to missing outcome data | Low |  |
| Bias in measurement of the outcome | NI | assesors were not blinded |
| Bias in selection of the reported result | Low |  |
|  | | |

| SYRCLE's risk of bias tool for animal studies | | | | | | |
| --- | --- | --- | --- | --- | --- | --- |
|  |  |  | **Yanatma, I (2020)** | **Fan Y, (2019)** | **Coleman, KM** | **Hwang, Y (2021)** |
| 1 | Selection bias | Sequence generation | High | High | NA | High |
| 2 | Selection bias | Baseline characteristics | Low | Low | High | High |
| 3 | Selection bias | Allocation concealment | High | High | NA | High |
| 4 | Performance bias | Random housing | Unclear | Unclear | Unclear | Unclear |
| 5 | Performance bias | Blinding | Unclear | Unclear | NA | Unclear |
| 6 | Detection bias | Random outcome assessment | Low | Low | Low | Low |
| 7 | Detection bias | Blinding | Unclear | Unclear | Low | Unclear |
| 8 | Attrition bias | Incomplete outcome data | Low | Low | Low | Low |
| 9 | Reporting bias | Selective outcome reporting | Low | Low | Low | Low |
| 10 | Other | Other sources of bias | Low/Unclear | Low/Unclear | High | Low/Unclear |

| Quality Assessment Tool For In Vitro Studies (QUIN Tool) |  |  |  |
| --- | --- | --- | --- |
|  | Wollina U, (2018) | Courderot-Masuyer, C, 2016 | Hwang, Y (2021) |
| Clearly stated aims objective | 2 | 2 | 2 |
| Detailed explanation of sample size calculation | 0 | 0 | 0 |
| Detailed explanation of sampling technique | 2 | 2 | 2 |
| Details of comparison group | NA | NA | NA |
| Detailed explanation of methodology | 2 | 2 | 2 |
| Operator details | 2 | 2 | 2 |
| Randomization | NA | NA | NA |
| Method of measurement of outcome | 2 | 2 | 2 |
| Outcome assessor details | 0 | 0 | 0 |
| Blinding | NA | NA | NA |
| Statistical analysis | 1 | 2 | 1 |
| Presentation of results | 1 | 2 | 2 |
|  | 0.6 | 0.7 | 0.65 |
| RISK OF BIAS | MEDIUM RISK OF BIAS | LOW RISK OF BIAS | MEDIUM RISK OF BIAS |
|  |  |  |  |
|  |  |  |  |
